# The bidirectional relationship of obesity and labor market status - Findings from a German prospective panel study

**DOI:** 10.1038/s41366-022-01105-3

**Published:** 2022-03-26

**Authors:** Hans Dietrich, Johannes Hebebrand, Volker Reissner

**Affiliations:** 1 Institute for Employment Research (IAB), Regensburger Str. 104, 90478 Nürnberg, Germany; 2 Department of Child and Adolescent Psychiatry and Psychotherapy, University of Duisburg-Essen, Wickenburgstr. 21, 45147 Essen, Germany

**Keywords:** Epidemiology, Risk factors

## Abstract

**Background:**

Given the inconsistent findings regarding associations between obesity and unemployment, our analysis is one of the few that explores bidirectional changes in obesity and unemployment. In our prospective study, we address factors associated with the

- transition into and transition out of obesity, including unemployment, and
- transition into and out of unemployment, including obesity.

**Subjects and methods:**

The Labor Market and Social Security-Panel (PASS) consists of two independent, nationally representative German subsamples: residents receiving unemployment benefits (50%) and a representative sample of residents (50%). The sample contains *N* = 11 361 observations between two measurement points three years apart of *N* = 8440 individuals participating in two or three waves between 2009 and 2015. We analyzed potential predictors of the transition in and out of obesity and unemployment, including health-related quality of life (HrQoL) and physical activity, using logistic regression models.

**Results:**

1. Transition into obesity: Unemployed participants had a higher probability of exhibiting a body mass index (BMI) ≥ 35 kg/m^2^ three years later (transition into obesity classes II and III; Exp(B) = 1.5).
2. Transition out of obesity: Unemployment did not predict transition out of obesity. Physical activity at least once weekly increased the probability of no longer having a BMI ≥ 35 kg/m^2^ three years later (Exp(B) = 2.0).
3. Transition into unemployment: Obesity was not associated with becoming unemployed three years later. Participants with a lower mental HrQoL were more likely to become unemployed (Exp(B) = 0.98).
4. Transition out of unemployment: Unemployed individuals reporting a BMI of 30–34.9 kg/m^2^ were less likely to leave unemployment (Exp(B) = 0.67). A better physical HrQoL was associated with a higher probability of leaving unemployment (Exp(B) = 1.01).

**Conclusions:**

Obesity does not predict future unemployment, but unemployed individuals with obesity have a lower probability of labor market re-entry. Unemployment increases obesity risk. Interactions between obesity and possible confounding variables and their effect on unemployment warrants further examination.

## Introduction

The results reported regarding the effect of obesity on individuals’ (long-term) unemployment risks are inconsistent [1, 2]. Several longitudinal studies based on large and representative samples did not find obesity to be a predictor of unemployment [3–7]. According to a Finnish birth cohort study including 9745 participants obesity at 14 years of age (body mass index [BMI] at or above the 95th percentile: males 23.7 kg/m^2^, females 23.8 kg/m^2^) did not predict long-term unemployment (total duration of unemployment in excess of 365 days over the twelve-year period prior to age 31) [3]. Similarly, Virtanen and colleagues (Sweden) found no effect of a BMI ≥ 25 kg/m^2^ at age 30 on labor market status during the following twelve years [4]. However, less than “good” self-rated health or a depressed mood predicted later unemployment. Neither a BMI between 25 kg/m^2^ and 30 kg/m^2^ nor a BMI ≥ 30 kg/m^2^ emerged as a significant predictor of transition to unemployment for employed workers with a mean age of 55.2 years (standard deviation [SD] 3.5 years) in a large European sample during the four-year follow-up [5]. These results are generally in accordance with the results of a meta-analysis by Robroeck et al., which did not show a direct correlation between obesity and later unemployment [6]. In addition, a more recent study by the same investigators did not identify obesity as a risk factor for unemployment within ten years for either male or female Dutch workers with a mean age of 39.0 years (SD 10.6) [7].

In contrast, more recent representative, large-scale longitudinal studies show gender-specific associations between obesity and later unemployment. According to both Paraponaris et al. [8] (French subjects, observation period of 20 years; deviation of >5 kg/m^2^ from the median BMI at age 20 served as a predictor) and Lindeboom et al. [9] (British subjects, observation period of ~20 years; respondents’ BMI and their parents’ BMI at baseline served as predictors), the effects of obesity were stronger for women. In a French prospective longitudinal study, obesity emerged as a significant predictor of incident unemployment for females over the four-year observation period (odds ratio: OR = 2.0; 95% confidence interval (CI) 1.2–3.4) [10]. Norrbäck and colleagues found severe effects of obesity and mobility disability on individuals’ unemployment risk in Sweden (adjusted for gender) [11].

Overall, more consistent results have been reported for the effect of unemployment on the development of obesity. A prospective study based on two English household panels (European Prospective Investigation of Cancer (EPIC) and British Household Panel Survey (BHPS)) analyzed changes in weight in relation to subjects’ labor market transitions into unemployment, retirement or employment maintenance [12]. That study controlled for initial body weight when using both data sets. According to the EPIC data, women with a mean age of 53.4 (SD 6.5) who became unemployed over the 3.5-year follow-up period gained more weight per year (0.7 kg/year) than women who stayed employed (0.49 kg/year). Combined BHPS data for males and females showed an association of job loss and weight gain (i.e., 1.56 kg/year in contrast to 0.6 kg/year in those who maintained their employment). Laitinen et al. reported a higher risk of obesity after longer periods of unemployment in women [3]. In a large Korean cross-sectional study, women with overweight or obesity showed a higher risk of unemployment than normal- or underweight women [13].

However, despite the rich but still inconsistent literature on the association of obesity and unemployment experience, there is still a lack of bidirectional studies. In accordance with the analytical study design of Laitinen et al., the current investigation is one of the few studies addressing the bidirectional association of obesity and labor market status [3]. We aimed to establish whether labor market status is a predictor of transition into obesity and, conversely, whether weight classes predict transitions into and out of unemployment within a three-year observation window. We addressed the following two research questions by employing a large-scale prospective population sample (Panel Labor Market and Social Security, PASS):

1. Associations of obesity (cutoffs: BMI ≥ 30 kg/m^2^, ≥35 kg/m^2^ and ≥40 kg/m^2^) with the transition into and out of unemployment,
2. Associations of unemployment status with the transition into and out of obesity (cutoffs: BMI ≥ 30 kg/m^2^, ≥35 kg/m^2^ and ≥40 kg/m^2^).

## Subjects and methods

In contrast to many other studies, we applied a short observation period approach, analyzing obesity or unemployment outcomes three years after a baseline measurement. Regarding transitions into or out of obesity, we established three BMI cutoffs according to the World Health Organization (WHO) classification [14]. In addition, with regard to transitions in and out of unemployment, we refer to self-reported unemployment versus other employment status. In addition to obesity or labor market status at baseline, we include further potential predictors suggested by the literature, such as subjective health measures and health behavior, former unemployment experience over the life course, obesity of household members and educational degrees.

### Study population

The data were obtained from the panel study “Labor Market and Social Security” (PASS). This is a representative household panel survey of the German residential population oversampling households receiving welfare benefits (UB-II subsample). The PASS panel was established by the Institute for Employment Research (IAB) in 2006 [15]. The PASS population consists of individuals nested in households.

Approximately 50% of the initial sample encompassed households receiving welfare benefits, and ~50% of the initial sample did not. Households were assigned to one of the two subpopulations only (population subsample or UB-II subsample) [16]. With respect to panel attrition, the sample population was refreshed in 2005 and 2011. Additionally, the welfare-receiving population was refreshed annually to respond to new household entrants into welfare recipiency [15]. Each household member above the age of 15 is addressed annually with an individual questionnaire [16].

The overall initial response rate at wave 1 (W1; 2007) at the household level was 30.1%, resulting in *N* = 12,794 households, including 18,972 individuals within *N* = 6804 households (*N* = 9586 individuals) for the representative population subsample and *N* = 5990 households (*N* = 9386 individuals) for the welfare benefit recipient subsample (UB-II) [17]. The panel continuity at the individual level was quite high for subsequent waves; for example, in wave 6, the response rate for participants in wave 5 was 85.7% [18].

### Study design

To address the bidirectional analyses of obesity and unemployment experience, we employed PASS data from waves 3, 6, and 9 only, at which the individual questionnaire of the PASS survey included an extended health module. The assessments of body height/weight and health-related behavior are part of this extended health module. We restricted our sample to individuals who participated in at least two consecutive PASS waves based on the extended health module. As three measurements over six years create a rather short longitudinal observational window, which severely limits the application of panel estimators and sample sizes of models for less prevalent degrees of obesity (BMI ≥ 35 kg/m^2^ and BMI ≥ 40 kg/m^2^), we applied transition models where we defined the first measurement point of each three-year period as T and the second measurement point (three years later) as T + 3. While all explanatory variables are observed in T, the corresponding outcome is reported in T + 3. Thus, and similar to Jusot et al. [10], we employed tuples out of wave 3 (2008/9) and wave six (2012) or wave six (2012) and wave nine (2015). As individuals could be part of both tuples, we calculated cluster-robust standard errors.

We restricted the age range to participants from 15 to 58 years at T to exclude age-related transitions into regular retirement at T + 3. Our data set includes 5117 observations from individuals participating in waves 3 and 6 and 6244 observations from individuals participating in waves 6 and 9. Overall, this results in 11,361 observations representing 8226 individuals available for our analysis. A total of 3037, 2080 and 3207 individuals participated in both tuples (w3/w6 and w6/w9), in tuple w3/w6 only, and in tuple w6/w9 only, respectively.

### Categorization of obesity groups and labor market status

We calculated individuals’ BMI according to the WHO definition based on self-reported information regarding height and weight. Both variables were assessed in PASS with an extended health module, which is applied every third wave/every three years. Based on the WHO classification of obesity, three BMI thresholds were assessed [14]:

- BMI ≥ 30.0 kg/m^2^ defining two groups, those below the cutoff of BMI ≥ 30.0 kg/m^2^ and those with BMI ≥ 30.0 kg/m^2^);
- BMI ≥ 35.0 kg/m^2^ defining two groups; and
- BMI ≥ 40.0 kg/m^2^ defining two groups.

We focused on up- or downward transitions based on these cutoffs. For this purpose, we estimated the relative risk for individuals’ BMI transitions crossing the respective cutoffs between T and T + 3 (BMI gain or BMI loss over the threshold, e.g., transition into BMI ≥ 30 kg/m^2^, BMI ≥ 35 kg/m^2^, BMI ≥ 40 kg/m^2^ or vice versa). We decided to apply this cutoff design in contrast to a dimensional BMI model to accommodate the WHO categorization of obesity into three BMI classes. These classes are frequently used to assess health risks [19–24].

From a bidirectional perspective, labor market transitions are the second outcome of interest. The PASS information regarding labor market transitions is based on self-reported information on an individual’s labor market status. As we are especially interested in transitions in and out of unemployment, we collapsed a broader list of labor market activities into the following seven status groups: unemployed (independent of registration status at the German Federal Employment Services), employed (including self-employed and dependently employed), student (in secondary, postsecondary and higher education or training), houseworker (including taking care of children or relatives at home), being on sick leave, early retirement (below the age of 60), and a residual group of others.

### Modeling

To address the bidirectional relationship of obesity and labor market status, we fitted four sets of exploratory multivariate models by applying logistic regression models:

- A first set of logit models was used to predict the transition above a defined BMI cutoff based on the three BMI cutoffs for class I–III obesity. The “transition into BMI ≥ 30 kg/m^2^” model included all participants who were below BMI 30 kg/m^2^ at T; the dependent variable indicated who became obese at T + 3. Analogously, we designed the models “transition into BMI ≥ 35 kg/m^2^” and “transition into BMI ≥ 40 kg/m^2^”. The three models assess the effect of the independent variables on the risk of transition based on the designated obesity cutoff values.
- A second set of logit models was used to predict the transition out of obesity based on our three obesity cutoffs. The baseline population consisted of individuals with BMI values above the respective cutoffs.
- A third set of stepwise logit models predicts the transition into unemployment. Here, the model baseline included all participants who were employed at T as the reference group. At T + 3_,_ they either remained employed or reported being unemployed at T + 3.
- A fourth set of stepwise logit models sought to address transitions out of unemployment: At baseline, all participants who reported unemployment at T were included, and the logit model estimated the individual risk of transitioning from unemployment to any other labor market status at T + 3.

### Predictors

In addition to obesity and labor market status, as key explanatory variables, we included a set of additional risk factors based on the literature review. We employed the health-related quality of life (HrQoL) measurement as a proxy for self-perceived health [25]. HrQoL was assessed with the SF-12, a reduced form of the 36-Item Short-Form Health Survey (SF-36) [26, 27]. A standardized scoring algorithm results in a score for the physical and mental component summaries [28]. Scores range from 0 to 100 points, with higher scores indicating better self-rated health. A meta-analysis of eight cross-sectional studies revealed lower scores for physical HrQoL in adults with a BMI ≥ 25 compared with those in normal-weight adults [29].

We additionally included smoking behavior and physical exercise activities as health behavior-related predictors. Smoking behavior was categorized as follows: never smoked, stopped smoking and smoking.

Self-rated physical exercise activities (“How often do you engage in active sports, fitness training or gymnastics?”) were measured on a five-point scale from “every day” to “never”. There is evidence regarding household member effects on obesity, especially in the case of younger and female subjects [30]; thus, we included information on the presence of additional subjects with obesity in the participant’s household [30].

As mentioned, the literature reports inconsistent findings regarding the effect of former unemployment experience on obesity, while there are consistent findings on the scarring effects of repeated unemployment experiences [31]. We included the number of unemployment years over the life course. As health- and obesity-related behaviors are related to social position or socioeconomic class, we controlled for the level of respondents’ education as a proxy for the respondents’ socioeconomic status. Finally, the literature review revealed gender-specific effects on the risks for both obesity and unemployment.

As further control variables, we used former unemployment experience over the life course, obesity of household members, migration background and region (old versus new German Länder). To capture the oversampling of the German UBII household population, we included a subsample dummy (UBII sample vs. population sample). The variable “wave” indicates the year of measurement point T. Due to the panel nature of our data, individuals could be included in both tuples. To control for repeated measurement of individuals, we applied clustered standard errors [32]. Three levels of significance were defined: *p* ≤ 0.05, *p* ≤ 0.01, and *p* ≤ 0.001.

PASS adhered to the ethical standards of the Institute for Employment Research. Ethical approval was not required for this secondary analysis of anonymized data performed using Stata 14 (Statacorp LLC College Station, Texas, USA).

## Results

### Description of the sample

The sociodemographic variables for the respondents from W3 indicated that *N* = 6162 (54.2%) were female. The mean age of the population was 39.0 years at W3 (see Supplementary Information Table 1). A total of 81.8% of employed and 76.7% of unemployed males reported a BMI < 30 kg/m^2^. The percentage of employed males with a BMI ≥ 35 kg/m^2^ (obesity classes II + III) was 3.9%, in contrast to 8.5% for unemployed males. Similar patterns emerged for the female population (see Supplementary Information Table 2).

Table 1 illustrates changes in WHO weight classes between T and T + 3. The majority of observations remained in the original weight classes (overall, 72.9%). In transitions from one weight class to the next, most respondents moved to the neighboring groups above or below. Of the participants with class I obesity at T, 61% of these observations indicated that these participants also remained in obesity class I at T + 3, 17.3% gained weight and moved to obesity class II, and 17.8% lost weight and moved from obesity class I to preobesity. Correspondingly, 56.4% of all individuals with obesity (class I) at T + 3 were already individuals with class I obesity at T.

**Table 1:** BMI development between T and T + 3, transitions in and out of BMI-classes (rows/columns are percentages of observations).

BMI class at T + 3							
BMI class at T	Underweight < 18.5	Normal weight 18.5–24.9	Preobesity 25–29.9	Obesity class I 30–34.9	Obesity class II 35–39.9	Obesity class III ≥ 40	Total
Outflows							
<18.5	46.72	51.37	1.09	0.55	0.00	0.27	100
18.5–24.9	2.01	80.53	16.44	0.79	0.11	0.11	100
25–29.9	0.09	13.22	71.15	14.09	1.17	0.27	100
30–34.9	0.07	1.54	17.78	61.01	17.34	2.27	100
35–39.9	0.23	0.68	4.51	26.64	51.47	16.48	100
≥40	0.00	0.00	4.66	5.51	19.07	70.76	100
Total	2.55	44.92	31.73	13.26	4.97	3.57	100
Inflows							
<18.5	60.00	3.75	0.11	0.14	0.00	0.35	3.28
18.5–24.9	38.25	86.97	25.13	2.91	1.08	2.09	48.41
25–29.9	1.05	8.80	67.01	31.76	7.03	3.14	29.88
30–34.9	0.35	0.42	6.86	56.35	42.70	10.80	12.25
35–39.9	0.35	0.06	0.56	7.97	41.08	25.44	3.97
≥40	0.00	0.00	0.31	0.88	8.11	58.19	2.11
Total	100.00	100.00	100.00	100.00	100.00	100.00	100.0

Notes: Chi^2^ = 1.9e4, *p* < 0.001; Source: PASS19.

### Predictors of transition into and out of obesity (BMI ≥ 30 kg/m^2^, ≥ 35 kg/m^2^ and ≥ 40 kg/m^2^)

We employed logistic regression models to explore variables associated with a BMI transition at the follow-up assessment. As we categorized obesity based on the three cutoffs used to define the WHO obesity classes (BMI: 30, 35 and 40), we calculated three models describing the transition into a higher class and three models describing the transition into a lower obesity class or into preobesity or normal weight.

As a baseline, we employed in the first model all observations below the BMI cutoff <30 kg/m^2^; in the second model, all observations below the BMI cutoff <35 kg/m^2^; and in the third model, all observations below the BMI cutoff <40 kg/m^2^ in T. Other restrictions were not applied. In addition to employment status, we included smoking behavior, physical exercise and HrQoL as potential predictor variables. Further control variables were also included (socioeconomic background, age, gender, region, subsample and wave).

While unemployment showed a positive but statistically insignificant effect on transiting the BMI-30 cutoff at T + 3, the odds ratios to cross the BMI cutoff of 35 kg/m² at T + 3 were 1.5 times greater for unemployed participants than for employed participants (reference) and close to twice as high for surpassing a BMI of 40 kg/m². Students were 0.4 times less likely to cross that obesity cutoff point than employed individuals. Better physical HrQoL was associated with a slightly lower probability of exceeding BMI ≥ 35 kg/m^2^ (*p* ≤ 0.001). Participants who had not engaged in physical exercise during the past three years showed a significantly higher risk of surpassing a BMI of 35 kg/m². The risk for developing a BMI ≥ 35 kg/m^2^ at follow-up was approximately seven times higher for participants living with other household members with obesity than for those who lived alone or with household members without obesity. With regard to the transition to BMI ≥ 35 kg/m^*2*^, *N* = 10,564 observations were included in the model (Table 2). Similar results were obtained for the models “transition to BMI ≥ 30 kg/m^2^” and “transition to BMI ≥ 40 kg/m^2^”.

**Table 2:** Predictors of transition into BMI ≥ 30 or ≥ 35 or ≥ 40 kg/m^2^.

Individual characteristics at T	Transition into OBE I + II + III (BMI ≥ 30 kg/m^2^)		Transition into OBE II + III (BMI ≥ 35 kg/m^2^)		Transition into OBE III (BMI ≥ 40 kg/m^2^)	
	OR	95%-CI	OR	95%-CI	OR	95%-CI
Employment status						
Employed (reference)	1.0000		1.0000		1.0000	
Unemployed	1.1435	(0.89–1.47)	1.4506*	(1.06–1.98)	1.9441**	(1.21–3.11)
Student	0.7343	(0.46–1.17)	0.3919**	(0.20–0.75)	0.5511	(0.17–1.77)
Housework	0.9025	(0.57–1.42)	1.4828	(0.88–2.49)	1.3622	(0.56–3.29)
Early retirement	0.8481	(0.45–1.58)	1.3423	(0.67–2.67)	2.0691	(0.80–5.37)
Other activities	0.7910	(0.48–1.30)	1.1853	(0.66–2.14)	1.7186	(0.74–3.98)
On sick leave	1.2497	(0.46–3.43)	0.9876	(0.20–4.82)	1.2611	(0.15–10.93)
Duration of unemployment experience (cumulated; years)	0.9924	(0.98–1.01)	0.9830	(0.96–1.01)	1.0067	(0.98–1.04)
Living with other obese people	19.5064***	(15.06–25.27)	7.0547***	(5.31–9.37)	5.4016***	(3.40–8.59)
Smoking behavior						
Never smoked	1.0964	(0.87–1.37)	1.2425	(0.94–1.65)	1.4526	(0.92–2.29)
Stopped smoking	1.4606**	(1.14–1.87)	1.3670	(0.99–1.89)	1.1393	(0.66–1.97)
Smoking (reference)	1.0000		1.0000		1.0000	
Physical exercise						
Several times per week (reference)	1.0000		1.0000		1.0000	
Once per week	1.1718	(0.88–1.57)	1.0106		0.9042	(0.45–1.83)
Less often	1.5230**	(1.18–1.96)	1.2993		1.3868	(0.79–2.44)
Never	1.5856***	(1.24–2.03)	1.5259**		1.9362*	1.16–3.23)
Health-related quality of life						
Physical component	0.9857**	(0.98–1.00)	0.9766***		0.9741**	(0.96–0.99)
Mental component	0.9970	(0.99–1.01)	1.0009		0.9978	(0.98–1.01)
Gender, female	0.8961	(0.74–1.09)	1.2237		1.6433*	(1.10–2.46)
Observations	9152		10564		10756	
Pseudo R^2^	0.125		0.112		0.114	

Notes: *p ≤ 0.05, **p ≤ 0.01, ***p ≤ 0.001; In parentheses: Upper and lower 95%-Confidence Intervals (CI); Source: PASS19; Clustered standard errors applied. Controls: human capital (years of schooling), marital status, age, migration background, region, wave, subsamples.

With regard to transitions out of BMI ≥ 35 kg/m^2^, we included all respondents above the respective BMI cutoff at T as the baseline (*N* = 707 observations associated with a BMI ≥ 35 kg/m^2^; Table 3). Physical activity at least once a week increased the probability of falling below this BMI cutoff point. The effect of physical activity was even more pronounced in the model for the transition out of BMI ≥ 40 kg/m^2^ (*N* = 221 observations). Participants with a lower mental HrQoL had a slightly and significantly lower BMI, and students had significantly higher probabilities of reducing their BMI below the cutoff point of BMI ≥ 30 kg/m^2^.

**Table 3:** Predictors of transition out of BMI ≥ 30 or ≥ 35 or ≥ 40 kg/m^2^.

Individual characteristics at T	Transition out of OBE I + II + III (BMI ≥ 30 kg/m^2^)		Transition out of OBE II + III (BMI ≥ 35 kg/m^2^)		Transition out of OBE III (BMI ≥ 40 kg/m^2^)	
	OR	95%-CI	OR	95%-CI	OR	95%-CI
Employment status						
Employed (reference)	1.0000		1.0000		1.0000	
Unemployed	0.9863	(0.70–1.38)	1.0938	(0.68–1.76)	1.0041	(0.43–2.37)
Student	2.6437**	(1.28–5.47)	1.3869	(0.44–4.37)	1.0000	
Housework	1.3941	(0.78–2.50)	0.9503	(0.36–2.48)	1.8849	(0.40–8.83)
Early retirement	1.1180	(0.58–2.15)	1.4192	(0.58–3.48)	1.7639	(0.45–6.95)
Other activities	1.2677	(0.64–2.49)	0.8053	(0.25–2.60)	0.6302	(0.07–5.54)
On sick leave	1.0696	0.21–5.32)	0.6064	(0.08–4.60)	1.0000	
Duration of unemployment experience (cumulated; years)	1.0107	(0.99–1.03)	0.9883	(0.96–1.02)	1.0120	(0.96–1.07)
Living with other obese people	0.6595*	(0.48–0.91)	1.0099	(0.65–1.57)	0.6903	(0.30–1.59)
Smoking behavior						
Never smoked	0.5747***	(0.42–0.79)	1.0108	(0.64–1.59)	0.8757	(0.39–1.99)
Stopped smoking	0.6168**	(0.44–0.87	1.3061	(0.80–2.14)	2.1921	(0.94–5.12)
Smoking (reference)	1.0000		1.0000		1.0000	
Physical exercise						
Several times per week (reference)	1.0000		1.0000		1.0000	
Once per week	1.4706	(0.97–2.24)	1.9647*	(1.01–3.84)	6.5694**	(1.69–25.48)
Less often	1.0040	(0.69–1.46)	1.3267	(0.76–2.33)	1.7570	(0.56–5.47)
Never	0.9345	(0.66–1.32)	0.8783	(0.53–1.45)	1.7948	(0.69–4.68)
Health-related quality of life						
Physical component	1.0107	(1.00–1.03)	1.0200*	(1.00–1.04)	1.0369	(1.00–1.08)
Mental component	0.9884*	(0.98–1.00)	1.0111	(1.00–1.03)	1.0258	(1.00–1.06)
Gender, female	0.9621	(0.72–1.28)	0.6857	(0.44–1.06)	0.9166	(0.39–2.16)
Observations	2119		707		221	
Pseudo R^2^	0.047		0.043		0.125	

Notes: *p ≤ 0.05, **p ≤ 0.01, ***p ≤ 0.001; In parentheses: Upper and lower 95%-Confidence Intervals (CI); Source: PASS19; Clustered standard errors applied.

Controls: human capital (years of schooling), marital status, age, migration background, region, wave, subsamples.

### Predictors of transition into and out of unemployment

We employed two logistic regression models to analyze the association of BMI with the outcome transition into unemployment. At baseline, we included all respondents who were not unemployed at T (Table 4). Here, BMI (categorized into six BMI classes according to the WHO) served as a key predictor, with normal weight (BMI: 18.5–25 kg/m^2^) as a reference. Estimations were made using two models. In addition to the factors included in Model 1 (BMI classes, health behavior, unemployment experience, living together with obese persons, gender, and further controls), we added physical and mental HrQoL in Model 2. Both models were based on *N* = 8374 observations and revealed robust results. The predictor variable BMI did not reach significance. Participants who never smoked or stopped smoking showed significantly lower odds of becoming unemployed at follow-up compared with smokers. Participants who never engaged in physical exercise were more likely to become unemployed than those who exercised several times per week. Longer durations of unemployment were associated with a higher unemployment risk. Women had a lower risk of becoming unemployed than males. Introducing mental HrQoL as an additional variable revealed that better mental health was associated with a slightly lower probability of becoming unemployed, while physical health showed no effect. Both models explain ~12% of the total variance. Likelihood-ratio tests indicated a small but significant model improvement when introducing HrQoL.

**Table 4:** Predictors of transition into unemployment.

Individual characteristics at T		Transition into unemployment			
		Model 1		Model 2	
		OR	95%-CI	OR	95%-CI
WHO BMI classification					
Underweight	Below 18.5	1.0082	(0.62–1.64)	0.9934	(0.61–1.62)
Normal weight	18.5–24.9 (reference)	1.0000		1.0000	
Preobesity	25.0–29.9	1.0083	(0.82–1.24)	1.0086	(0.82–1.24)
Obesity class I	30.0–34.9	1.1059	(0.82–1.48)	1.1032	(0.82–1.48)
Obesity class II	35.0–39.9	0.6813	(0.41–1.14)	0.6735	(0.40–1.14)
Obesity class III	40.0 and above	1.5565	(0.88–2.75)	1.5169	(0.85–2.72)
Duration of unemployment experience (total number of years)		1.0205*	(1.00–1.04)	1.0180*	(1.00–1.03)
Household composition	Living with other obese people	1.2637	(0.90–1.77)	1.2620	(0.90–1.77)
Smoking behavior	Never smoked	0.5205***	(0.42–0.64)	0.5242***	(0.43–0.65)
	Stopped smoking	0.6012***	(0.46–0.78)	0.5990***	(0.46–0.78)
	Smoking	1.0000		1.0000	
Physical exercise					
	Several times a week	1.0000		1.0000	
	Once a week	0.8615	(0.65–1.14)	0.8496	(0.64–1.13)
	Less often	1.1869	(0.93–1.52)	1.1642	(0.91–1.49)
	Never	1.3073*	(1.03–1.66)	1.2737*	(1.00–1.62)
Health-related quality of life	Physical component			0.9976	(0.99–1.01)
	Mental component			0.9859***	(0.98–0.99)
Gender	Female	0.8384	(0.70–1.01)	0.8136*	(0.68–0.98)
	Observations	8374		8374	
	Pseudo *R*^2^	0.117		0.120	

Notes: *p ≤ 0.05, **p ≤ 0.01, ***p ≤ 0.001; Source: PASS19; In parentheses: Upper and lower 95%-Confidence Intervals (CI); Clustered standard errors applied.

Controls: human capital (years of schooling), marital status, age, migration background, region, wave, subsamples.

Two models were calculated regarding the outcome variable transition out of unemployment (Table 5). All respondents unemployed at T were introduced to Models 1 and 2 as baseline. Similar to the preceding models, we additionally included the HrQoL physical and mental components in Model 2. The explanatory variables corresponded with the preceding set of models. The analyses were based on *N* = 2897 observations for Models 1 and 2. In Model 1, unemployed participants with a BMI between 30 and 34.9 kg/m^2^ and those with a BMI between 35 and 39.9 kg/m^2^ at baseline had a lower risk of leaving unemployment. In Model 2, better physical HrQoL was associated with a higher probability of leaving unemployment. In Model 2, however, only a BMI between 30 and 34.9 kg/m^2^ was associated with a lower probability of leaving unemployment. A BMI between 35 and 39.9 kg/m^2^ was no longer predictive after the inclusion of the physical and mental quality of life variables in Model 2.

**Table 5:** Predictors of transition out of unemployment.

Individual characteristics at T		Transition out of unemployment			
		Model 1		Model 2	
		OR	95%-CI	OR	95%-CI
WHO BMI classification					
Underweight	Below 18.5	1.1628	(0.77–1.77)	1.1864	(0.77–1.83)
Normal weight	18.5–24.9 (reference)	1.0000		1.0000	
Preobesity	25.0–29.9	0.8877	(0.74–1.07)	0.8983	(0.74–1.08)
Obesity class I	30.0–34.9	0.6410***	(0.50–0.83)	0.6651**	(0.52–0.86)
Obesity class II	35.0–39.9	0.6545*	(0.46–0.94)	0.7051	(0.49–1.01)
Obesity class III	40.0 and above	0.7098	(0.46–1.09)	0.7768	(0.51–1.19)
Duration of unemployment experience (total number of years)		0.9758***	(0.96–0.99)	0.9763***	(0.96–0.99)
Household composition	Living with other obese people	1.1422	(0.84–1.56)	1.1644	(0.86–1.57)
Smoking behavior	Never smoked	1.0976	(0.91–1.33)	1.0925	(0.90–1.32)
	Stopped smoking	1.3674**	(1.10–1.71)	1.3797**	(1.11–1.72)
	Smoking	1.0000		1.0000	
Physical exercise					
	Several times a week	1.0000		1.0000	
	Once a week	1.1587	(0.88–1.52)	1.1581	(0.88–1.52)
	Less often	1.0194	(0.82–1.27)	1.0250	(0.82–1.28)
	Never	0.6907***	(0.57–0.84)	0.6977***	(0.57–0.85)
Health-related quality of life	Physical component			1.0123**	(1.00–1.02)
	Mental component			1.0044	(1.00–1.01)
Gender	Female	1.3034**	(1.10–1.54)	1.3062**	(1.11–1.54)
	Observations	2.897		2.897	
	Pseudo *R*^2^	0.062		0.065	

Notes: *p ≤ 0.05, **p ≤ 0.01, ***p ≤ 0.001; In parentheses: Upper and lower 95%-Confidence Intervals (CI); Source: PASS19; Clustered standard errors applied.

Controls: human capital (years of schooling), marital status, age, migration background, region, wave, subsamples.

In both models, longer unemployment exposure reduced the odds of leaving unemployment. Participants who gave up smoking had a higher probability of leaving unemployment than those who were still smoking. Participants who never engaged in physical exercise showed a lower probability of leaving unemployment than those who were physically active several times per week. Male sex was associated with a higher probability of leaving unemployment. Both models accounted for ~6% of the variance. Likelihood-ratio tests confirm a limited but significant model improvement when including HrQoL.

## Discussion

This investigation is one of the few studies to explore the bidirectional association of unemployment status with obesity and vice versa given a baseline assessment and observed outcomes in a follow-up assessment three years later, employing data from a representative prospective panel design (PASS panel). Our study did not show that employed participants with obesity at baseline had a higher probability of becoming unemployed at follow-up compared with employed subjects without obesity. However, our study revealed that among the participants who were unemployed at baseline, individuals with obesity showed a lower probability of exiting unemployment three years later than unemployed individuals without obesity. In general, our results are in accordance with some of the literature that states a positive effect of unemployment on weight gain. We did not find a significant effect of unemployment status on weight loss.

In detail, our analyses of panel data did not reveal an association between obesity at baseline and being unemployed at follow-up for those who were employed at the first measurement (transition into unemployment) under control of health-related behavior and cumulative unemployment duration. However, unemployed participants with obesity had lower chances of re-entering the labor market (transition out of unemployment; strong effect size and significance for BMI obesity classes I or II at baseline). Upon additionally controlling for self-reported physical HrQoL, the variable “Obesity class II” was no longer a significant predictor, indicating a possible mediating effect of physical HrQoL. We conclude that more research is required to elucidate the possible direct and indirect effects of obesity on individuals’ labor market status.

As the empirical evidence for the association of obesity and transitions in and out of unemployment is still inconsistent, our findings regarding the transition into unemployment are in accordance with those studies that reported an effect of obesity on the transition out of unemployment [8, 9]. However, we found no specific risks for employed participants with obesity who were unemployed three years later.

Similar to the analysis in our study, the analysis of longitudinal data from a French national health survey suggested that low self-rated health is a precursor for later unemployment [10]. In accordance with Virtanen et al. and Robroek et al., we found an association between self-rated health and unemployment [4, 6].

Our results demonstrate the importance of mental and physical HrQoL and possible challenges in detecting its association with obesity and employment status. The first challenge pertains to the measurement of self-reported health. In population-based studies, self-reported health is commonly assessed by a single-item general question asking individuals to rate their overall health. This very broad conceptualization is criticized as being dependent on many contexts such that individual comparability is often not possible, which may be one reason for the contrasting study results [33, 34]. It can be assumed that the SF-12 used in the PASS panel is a more suitable instrument that defines self-reported health more precisely than a single-item questionnaire.

The second challenge concerns potential mediator variables. Variables such as HrQoL, job insecurity, or physical activity may influence the relationship between BMI and self-reported health. Findings from the literature suggest that job insecurity adds to psychological burden, reduces self-reported health and leads to an increase in BMI [35–37]. As a source of social reinforcement, peer status is associated with dieting and other weight-related behaviors and cognitions [38]. Physical activity is positively associated with self-rated health and negatively associated with BMI depending on gender or ethnicity [39–41].

Taken together, our models suggest that a BMI ≥ 30 (≥35 or 40) kg/m^2^ is relevant for employment status. However, this effect is mediated by reduced physical and/or mental health. We hypothesize that obesity is not itself a barrier for staying in the labor market but that obese individuals’ perception of physical and mental fitness represents the barrier to active job searching and related activities necessary to enter the labor market [41].

With regard to the transition to a higher BMI cutoff, participants who experienced job loss were at higher risk of BMI ≥ 35 kg/m^2^ or ≥40 kg/m^2^ at follow-up, which is in accordance with UK panel data obtained by Monsivais and colleagues [12]. In contrast to the UK study, which did not reveal a contribution of physical activity to weight gain, the PASS data showed a higher risk of becoming obese for those who never engaged in physical activity. The associations between physical HrQoL and obesity groups were significant in all models, supporting the findings of Kolotkin and Andersen [42].

The probability of gaining weight is 5- to 19-fold higher for participants living with an obese partner than for those who do not. This finding is in accordance with studies reporting that an increase in spouses’ BMI also led to an increase in men’s and women’s BMI [43, 44]. Living with an obese individual may be associated with an unhealthy lifestyle and less physical activity [45].

As in another study, post-nicotine cessation-related weight gain was observed in the PASS data but only for the ‘transition to BMI ≥ 30 kg/m^2^’ model [46]. We found no significant gender effects except for the transition to BMI ≥ 40 kg/m^2^.

With regard to the transition out of obesity, a comparison of the three models resulted in a less consistent view. Participants who engaged in physical exercise showed a higher probability of reducing their BMI below 35 kg/m^2^ or 40 kg/m^2^. As expected, unemployment was not associated with weight loss or transition out of obesity status.

Our use of a categorical classification of BMI data may have led to a loss of power (reduced data level). Thus, we additionally deployed a linear OLS model to explore associations with relative BMI changes in the population (relative BMI change was calculated as [100* (BMI_(T)_ – BMI_(T-3)_)] / BMI_(T))_). This robustness test revealed similar results to those obtained from the categorical models describing the transition into or out of obesity (Supplementary Information Table 3).

Limitations of the study are related to the PASS panel data. Similar to other authors (Laitinen et al., 2002; Jusot et al., 2008), we employed predictors only from the baseline measurement to explain weight or labor market status three years later [3, 10]. This limitation is a result of our short panel duration of obesity measurements (three waves).

The oversampling of UBII households enabled both a representative sample of the German population and a sufficiently high number of observations of unemployment status and BMI-related status transitions.

As panel attrition may affect the estimates, all variables entered in the substantial models are assessed by selection models to test for attrition effects on the outcome of interest. Sensitivity analyses were performed for attrition between W3 and W6 and between W6 and W9. The results indicated only weak attrition effects. The duration of individuals’ unemployment experience and the type of subsample (UBII sample versus population sample) affect the attrition risk between W3 and W6. Moreover, the type of subsample and individuals’ labor market status (being sick as a reason for not working) affected the attrition rate between W6 and W9. There were no significant effects of age, gender, education, labor market status, or BMI.

Other sources of bias may be due to unobserved factors. For instance, on an individual level, motivation for job search activity may change with the duration of unemployment. On a macroeconomic level, the decrease in unemployment rates in Germany between 2009 (8.4%) and 2015 (6.4%) had an overall positive effect on the probability of finding a job, and even UBII beneficiaries benefitted from this economic effect [47]. Our panel study analyzed three-year periods, and individuals gained on average 2.3 kg over three years but with marginal gender or labor market status differences. In contrast, Monsivais et al. reported for a comparatively older population (EPIC) a weight increase of 0.7 kg per year for unemployed women, in contrast to 0.49 kg per year for employed women. The increase in weight in the male status group was less pronounced [12]. These differences may indicate the importance of specific sample characteristics and warrant additional attention.

Another limitation of this study may be the focus on status transitions. In this study, we did not analyze the simultaneous dynamics of the labor market and BMI status, which is a potential direction for future research.

## Conclusions

Future research is warranted to better assess the interaction between obesity and possible mediating variables and their effect on unemployment status. Counseling programs for unemployed individuals should focus on avoiding weight gain and related health problems. Counseling of obese individuals who are potentially first-time entrants in the labor market should target the reduction of obesity and related disease burden as well as other potential moderators, such as participants’ self-efficacy beliefs, to become successfully integrated into the labor market.

## Supplementary information

Supplementary Material: Table 1

Supplementary Material: Table 2

Supplementary Material: Table 3
