# Supplementary material for: The bidirectional relationship of obesity and labor market status - Findings from a German prospective panel study
Source: Int J Obes (Lond). 2022 Mar 26;46(7):1295–303. doi: 10.1038/s41366-022-01105-3 (PMC9239903; doi:10.1038/s41366-022-01105-3)
Supplement: Supplementary file 1 — Supplementary Material: Table 1 [file 41366_2022_1105_MOESM1_ESM.docx]

**Supplementary information Table 1: Sociodemographic sample characteristics and controls by WHO obesity class and sex at T (chi-square test for obesity class differences)**

| **Males** |  | |  | |  | |  | |  | |  | | **Females** | |  | |  | |  | |  | |  |
| --- | --- | --- | --- | --- | --- | --- | --- | --- | --- | --- | --- | --- | --- | --- | --- | --- | --- | --- | --- | --- | --- | --- | --- |
| **Obesity class** | **BMI< 30** | | **30≤**  **BMI< 35** | | **35≤**  **BMI< 40** | | **BMI≥ 40** | | **Total** | |  | | **Obesity class** | | **BMI< 30** | | **30≤**  **BMI< 35** | | **35≤**  **BMI< 40** | | **BMI≥ 40** | | **Total** |
|  |  | |  | |  | |  | |  | |  | |  | |  | |  | |  | |  | |  |
| **N (observations)** | 4.257 | | 681 | | 185 | | 76 | | 5,199 | |  | | N | | 4,968 | | 745 | | 278 | | 171 | | 6,162 |
| [%] | 81.88 | | 13.1 | | 3.56 | | 1.46 | | 100 | |  | | [%] | | 80.62 | | 12.09 | | 4.51 | | 2.78 | | 100 |
|  |  | |  | |  | |  | |  | |  | |  | |  | |  | |  | |  | |  |
| **Age ^***^** |  | |  | |  | |  | |  | |  | | **Age ^***^** | |  | |  | |  | |  | |  |
| ≤ 20 y. | 96.92 | | 2.64 | | 0.22 | | 0.22 | | 100 | |  | | ≤ 20 y. | | 95.54 | | 3.29 | | 0.7 | | 0.47 | | 100 |
| 20––29.9 y. | 84.11 | | 11.43 | | 3.37 | | 1.09 | | 100 | |  | | 20–29.9 y. | | 84.88 | | 9.7 | | 2.86 | | 2.55 | | 100 |
| 30–39.9 y. | 82.79 | | 13.35 | | 3.17 | | 0.69 | | 100 | |  | | 30–39.9 y. | | 81.84 | | 11.38 | | 4.3 | | 2.48 | | 100 |
| 40–49.9 y. | 79.59 | | 14.25 | | 4.11 | | 2.05 | | 100 | |  | | 40–49.9 y. | | 78.54 | | 12.61 | | 5.59 | | 3.26 | | 100 |
| 50–50.9 y. | 76.8 | | 16.57 | | 4.56 | | 2.07 | | 100 | |  | | 50–50.9 y. | | 74.78 | | 16.5 | | 5.5 | | 3.23 | | 100 |
|  |  | |  | |  | |  | |  | |  | |  | |  | |  | |  | |  | |  |
| **Migration status** | | |  | |  | |  | |  | |  | | **Migration status** | | | |  | |  | |  | |  |
| German | 82 | | 12.89 | | 3.34 | | 1.42 | | 100 | |  | | German | | 80 | | 12.25 | | 4.6 | | 2.92 | | 100 |
| Migration 1st generation | 79.13 | | 15.42 | | 4.52 | | 0.93 | | 100 | |  | | Migration 1st generation | | 82.57 | | 12.13 | | 2.94 | | 2.36 | | 100 |
| Migrated 2nd generation | 80.83 | | 11.98 | | 4.58 | | 2.61 | | 100 | |  | | Migrated 2nd generation | | 80.58 | | 10.56 | | 6.3 | | 2.56 | | 100 |
| No information | 84.4 | | 12.06 | | 2.13 | | 1.42 | | 100 | |  | | No information | | 81.75 | | 13.14 | | 3.65 | | 1.46 | | 100 |
|  |  | |  | |  | |  | |  | |  | |  | |  | |  | |  | |  | |  |
| **Academic degree ^***^** | | |  | |  | |  | |  | |  | | **Academic degree ^***^** | | | |  | |  | |  | |  |
| In school | 98.31 | | 1.69 | | 0 | | 0 | | 100 | |  | | In school | | 97.55 | | 2.45 | | 0 | | 0 | | 100 |
| Low | 67.66 | | 23.35 | | 6.59 | | 2.4 | | 100 | |  | | Low | | 69.64 | | 16.96 | | 5.8 | | 7.59 | | 100 |
| Medium | 79.85 | | 14.4 | | 4.12 | | 1.64 | | 100 | |  | | Medium | | 78.62 | | 13.31 | | 5.17 | | 2.9 | | 100 |
| High | 88.5 | | 8.96 | | 1.57 | | 0.97 | | 100 | |  | | High | | 90.03 | | 6.65 | | 1.66 | | 1.66 | | 100 |
|  |  | |  | |  | |  | |  | |  | |  | |  | |  | |  | |  | |  |
| **Marital status ^***^** | | |  | |  | |  | |  | |  | | **Marital status ^***^** | | | |  | |  | |  | |  |
| Not married | 83.99 | | 11.09 | | 3.49 | | 1.43 | | 100 | |  | | Not married | | 81.8 | | 11.16 | | 4.19 | | 2.85 | | 100 |
| Married | 78.98 | | 15.86 | | 3.66 | | 1.51 | | 100 | |  | | Married | | 79.21 | | 13.21 | | 4.9 | | 2.68 | | 100 |
| **Subsamples** | |  |  |  | |  | |  | |  | | **Subsamples** | |  | |  | |  | |  | |  | |
| SGBII sample | | 80.26 | 13.46 | 4.28 | | 1.99 | | 100 | |  | | SGBII sample | | 77.9 | | 13.5 | | 5.28 | | 3.32 | | 100 | |
| Population sample | | 84.03 | 12.62 | 2.6 | | 0.76 | | 100 | |  | | Population sample | | 84.49 | | 10.09 | | 3.42 | | 2 | | 100 | |
| **Region** | |  |  |  | |  | |  | |  | | **Region** | |  | |  | |  | |  | |  | |
| Old German Länder | | 81.96 | 12.78 | 3.79 | | 1.47 | | 100 | |  | | Old German Länder | | 81.53 | | 11.5 | | 4.21 | | 2.76 | | 100 | |
| New German Länder | | 81.69 | 13.83 | 3.03 | | 1.45 | | 100 | |  | | New German Länder | | 78.31 | | 13.6 | | 5.28 | | 2.81 | | 100 | |
|  |  | |  | |  | |  | |  | |  | |  | |  | |  | |  | |  | |  |
| Source: PASS19 | | | | | | | | | | | | | | | | | | | | | | | |
