# Supplementary material for: The bidirectional relationship of obesity and labor market status - Findings from a German prospective panel study
Source: Int J Obes (Lond). 2022 Mar 26;46(7):1295–303. doi: 10.1038/s41366-022-01105-3 (PMC9239903; doi:10.1038/s41366-022-01105-3)
Supplement: Supplementary file 2 — Supplementary Material: Table 2 [file 41366_2022_1105_MOESM2_ESM.docx]

**Supplementary Information Table 2: Descriptive statistics for the explanatory variables by WHO obesity class and sex at T (chi-square test for obesity class differences)**

|  |  |  |  |  |  |  |  |  |  |  |  |  |
| --- | --- | --- | --- | --- | --- | --- | --- | --- | --- | --- | --- | --- |
| **Obesity class:** | **BMI< 30** | **30≤**  **BMI< 35** | **35≤**  **BMI< 40** | **BMI≥ 40** | **Total** |  | **Obesity class:** | **BMI< 30** | **30≤**  **BMI< 35** | **35≤**  **BMI< 40** | **BMI≥ 40** | **Total** |
|  |  |  |  |  |  |  |  |  |  |  |  |  |
|  | **… for** | **males** |  |  |  |  |  | **… for** | **females** |  |  |  |
| **N (observations)** | 4,257 | 681 | 185 | 76 | 5,199 |  | **N (observations)** | 4,968 | 745 | 278 | 171 | 6,162 |
|  | 81.88 | 13.1 | 3.56 | 1.46 | 100 |  |  | 80.62 | 12.09 | 4.51 | 2.78 | 100 |
| **Employment status** |  |  |  |  |  |  | **Employment status** |  |  |  |  |  |
| Employed | 81.71 | 14.38 | 3.11 | 0.8 | 100 |  | Employed | 82.38 | 11.49 | 4.08 | 2.06 | 100 |
| Unemployed | 76.72 | 14.83 | 5.73 | 2.72 | 100 |  | Unemployed | 73.17 | 15.68 | 6.03 | 5.12 | 100 |
| At school/student | 95.62 | 4.25 | 0.14 | 0 | 100 |  | At school/student | 93.82 | 3.68 | 2.21 | 0.29 | 100 |
| Homework | 83.33 | 8.33 | 4.17 | 4.17 | 100 |  | Homework | 80.36 | 13.04 | 4.46 | 2.15 | 100 |
| Early retirement (pension) | 65.13 | 19.08 | 8.55 | 7.24 | 100 |  | Early retirement (pension) | 61.81 | 20.83 | 9.03 | 8.33 | 100 |
| Other activities | 88.57 | 9.52 | 0 | 1.9 | 100 |  | Other activities | 82.47 | 11.78 | 4.02 | 1.72 | 100 |
| On sick leave | 64.52 | 16.13 | 12.9 | 6.45 | 100 |  | On sick leave | 71.43 | 19.05 | 4.76 | 4.76 | 100 |
| **Unemployment duration** (mean of cumulated; years) | 0.62 | 0.93 | 1.42 | 2.32 |  |  | **Unemployment duration** (mean of cumulated; years) | 2.66 | 4.11 | 5.36 | 2.30 |  |
|  |  |  |  |  |  |  |  |  |  |  |  |  |
| **Living with obese person** |  |  |  |  |  |  | **Living with obese person** |  |  |  |  |  |
| No | 86.06 | 10.36 | 2.5 | 1.07 | 100 |  | No | 84.65 | 9.81 | 3.4 | 2.13 | 100 |
| Yes | 36.88 | 42.53 | 14.93 | 5.66 | 100 |  | Yes | 34.28 | 38.34 | 17.24 | 10.14 | 100 |
|  |  |  |  |  |  |  |  |  |  |  |  |  |
| **Smoking behavior** |  |  |  |  |  |  | **Smoking behavior** |  |  |  |  |  |
| Never smoked | 85.77 | 9.9 | 3.37 | 0.96 | 100 |  | Never smoked | 80.62 | 12.28 | 4.34 | 2.76 | 100 |
| Stopped smoking | 74.23 | 18.58 | 5.19 | 2 | 100 |  | Stopped smoking | 76.77 | 13.97 | 5.68 | 3.58 | 100 |
| Smoking | 82.05 | 13.31 | 3.01 | 1.63 | 100 |  | Smoking | 82.55 | 10.92 | 4.13 | 2.39 | 100 |
|  |  |  |  |  |  |  |  |  |  |  |  |  |
| **Physical exercise** |  |  |  |  |  |  | **Physical exercise** |  |  |  |  |  |
| At least once a week | 89.56 | 7.98 | 1.84 | 0.61 | 100 |  | At least once a week | 85.19 | 9.67 | 3.38 | 1.76 | 100 |
| Never | 74.8 | 16.12 | 6.01 | 3.07 | 100 |  | Never | 85.22 | 10.49 | 3.05 | 1.24 | 100 |
|  |  |  |  |  |  |  |  |  |  |  |  |  |
| **to be continued** | | | | | | | | | | | | |

**Supplementary Information Table 2: Descriptive statistics for the explanatory variables by WHO obesity class and sex at T (chi-square test for obesity class differences; continued)**

|  |  |  |  |  |  |  |  |  |  |  |  |  |
| --- | --- | --- | --- | --- | --- | --- | --- | --- | --- | --- | --- | --- |
| **Obesity class:** | **BMI< 30** | **30≤**  **BMI< 35** | **35≤**  **BMI< 40** | **BMI≥ 40** | **Total** |  | **Obesity class:** | **BMI< 30** | **30≤**  **BMI< 35** | **35≤**  **BMI< 40** | **BMI≥ 40** | **Total** |
|  |  |  |  |  |  |  |  |  |  |  |  |  |
|  | **… for** | **males** |  |  |  |  |  | **… for** | **females** |  |  |  |
| **Health-related**  **quality of life** |  |  |  |  |  |  | **Health-related**  **quality of life** |  |  |  |  |  |
| Physical | 52.86 | 49.46 | 47.24 | 39.77 |  |  | Physical | 51.97 | 47.54 | 46.08 | 40.74 |  |
| Mental | 53.81 | 52. 79 | 53.81 | 52.80 |  |  | Mental | 51.61 | 51.33 | 50.43 | 49.03 |  |
|  |  |  |  |  |  |  |  |  |  |  |  |  |
| Notes: ^*^ p ≤ 0.05. ^**^ p ≤ 0.01. ^***^ p ≤ 0.001  Source: PASS19 | | | | | | | | | | | | |
