# Supplementary material for: The bidirectional relationship of obesity and labor market status - Findings from a German prospective panel study
Source: Int J Obes (Lond). 2022 Mar 26;46(7):1295–303. doi: 10.1038/s41366-022-01105-3 (PMC9239903; doi:10.1038/s41366-022-01105-3)
Supplement: Supplementary file 3 — Supplementary Material: Table 3 [file 41366_2022_1105_MOESM3_ESM.docx]

**Supplementary information Table 3: Predictors of BMI change between T and T+3**

| **Individual characteristics at T** |  | BMI-change |  |  |  |  |
| --- | --- | --- | --- | --- | --- | --- |
|  |  | Coefficient |  | 95%-CI | |  |
| **WHO BMI classification** |  |  |  | Lower | Upper |  |
| Underweight | Below 18.5 | 6.2553 | *** | 4.7174 | 7.7932 |  |
| Normal weight (reference) | 18.5–24.9 | 0.0000 |  |  |  |  |
| Preobesity | 25.0–29.9 | 1.7044 | *** | 1.2758 | 2.1329 |  |
| Obesity class I | 30.0–34.9 | -1.2142 | *** | -1.8728 | -0.5556 |  |
| Obesity class II | 35.0–39.9 | -3.3998 | *** | -4.5465 | -2.2531 |  |
| Obesity class III | 40.0 and above | -7.3705 | *** | -9.2670 | -5.4740 |  |
|  |  |  |  |  |  |  |
| **Employment status** |  |  |  |  |  |  |
|  | Employed (reference) | 0.0000 |  |  |  |  |
|  | Unemployed | 0.1006 |  | -0.4270 | 0.6281 |  |
|  | Student | -0.9592 | * | -1.7973 | -0.1211 |  |
|  | Housework | -0.9097 | * | -1.6661 | -0.1534 |  |
|  | Early retirement | -1.7208 | * | -3.1339 | -0.3078 |  |
|  | Other activities | -0.1713 |  | -1.1163 | 0.7737 |  |
|  | On sick leave | -0.6900 |  | -2.8807 | 1.5007 |  |
|  |  |  |  |  |  |  |
| **Duration of unemployment experience (total number of month)** |  | -0.0009 |  | -0.0051 | 0.0034 |  |
| **Household composition** | Living with other obese people | 3.4885 | *** | 2.5297 | 4.4473 |  |
|  |  |  |  |  |  |  |
|  |  |  |  |  |  |  |
| **Smoking behavior** | Never smoked | 0.4109 |  | -0.0334 | 0.8551 |  |
|  | Stopped smoking | 0.1589 |  | -0.3140 | 0.6318 |  |
|  | Smoking | 0.0000 |  |  |  |  |
|  |  |  |  |  |  |  |
| **Physical exercise** | Several times a week | -0.7473 | ** | -1.2261 | -0.2685 |  |
|  | Once a week | -0.8504 | ** | -1.4713 | -0.2294 |  |
|  | Less often | -0.3918 |  | -0.9241 | 0.1404 |  |
|  | Never | 0.0000 |  |  |  |  |
|  |  |  |  |  |  |  |
| **Health-related** | Physical component | -0.0091 |  | -0.0288 | 0.0106 |  |
| **quality of life** | Mental component | -0.0111 |  | -0.0289 | 0.0066 |  |
|  |  |  |  |  |  |  |
| **Gender:** Female |  | 0.1371 |  | -0.2212 | 0.4954 |  |
|  |  |  |  |  |  |  |
|  |  |  |  |  |  |  |
| Constant |  | 3.9993 |  | 2.1543 | 5.8443 |  |
| Number of observations | 11 072 |  |  |  |  |  |
| Number of individuals | 8 121 |  |  |  |  |  |
| Adj. R2 | 0.056 |  |  |  |  |  |
|  |  |  |  |  |  |  |

Notes: OLS regression coefficients; ^*^ p ≤ 0.05. ^**^ p ≤ 0.01. ^***^ p ≤ 0.001; Clustered standard errors applied; Source: PASS19; Controls: human capital (years of schooling). marital status. age. migration background. region. wave. subsamples; Dependent variable: Relative BMI-change = [100*(BMI_(T)_ – BMI_(T-3)_)]/ BMI_(T)_).
